# Generalized Seizure in a Mauritian Woman Taking Bupropion

**DOI:** 10.1371/journal.pmed.0010015

**Published:** 2004-10-19

**Authors:** Marie France Lan Cheong Wah, Lan Sem Hing Lan Cheong Wah

**Affiliations:** **1**Faculty of Science, University of MauritiusReduitMauritius; **2**Port-LouisMauritius

## Abstract

A 24-y-old woman was admitted to the emergency department having had a generalized seizure (acute loss of consciousness, convulsive movements of her arms and legs, and confusion on regaining consciousness). She was on the sixth day of treatment with 300 mg daily of slow-release bupropion (Zyban SR) as an aid to smoking cessation. She had a past medical history of tonsillectomy and hay fever, for which she was taking budesonide nasal drops (two drops daily, each drop 200 mcg). She was on no other medication. There was no history of head trauma, liver disease, or alcohol withdrawal. Clinical examination, including neurological examination, was normal. The patient's weight was 48 kg. Her blood pressure was 130/80 mm Hg. Electrocardiogram showed a sinus tachycardia at 102 beats per minute. Radiography of the skull and a computed tomography scan of the brain without contrast were both normal. The patient's blood glucose, urea, electrolytes, and liver function tests were all normal. Her serum calcium was 2.01 mmol/l (normal range, 2.0–2.6 mmol/l) and her hemoglobin was 116 g/l (normal range, 120–140 g/l). The bupropion was discontinued, and the patient recovered without any further seizures or other neurological sequelae.

## Abstract 2

Bupropion recently came onto the Mauritian market as an aid to smoking cessation. This case report is a useful reminder to clinicians of the risks of taking the drug

## Case Discussion

### Bupropion for Smoking Cessation

Originally developed as an antidepressant, bupropion has more recently been licensed in many countries as an aid to smoking cessation. It came onto the Mauritian market as a smoking cessation aid in November 2003.

The British National Formulary (www.bnf.org) recommends starting the drug one to two weeks before the target smoking stop date, initially at a dose of 150 mg daily for 6 d and then 150 mg twice daily. The maximum period of treatment is 7–9 wk; treatment should be discontinued if abstinence is not achieved by 7 wk.

The efficacy of bupropion as an aid to smoking cessation has been shown in randomized, double-blind, placebo-controlled trials [1,2]. But there have also been reports of death, seizure, serum sickness, generalized acute urticaria, myocardial infarction, and psychosis in people taking the drug [3,4,5,6,7]. Our report is of a woman who had a generalized seizure on the sixth day of treatment with bupropion; she had no other risk factors for seizures.

### Contraindications to Bupropion

To reduce the risk of seizures, the drug should not be given to patients with a current seizure disorder or any history of seizures, with a current or previous diagnosis of bulimia or anorexia nervosa, with a known central nervous system tumour, or to those experiencing abrupt withdrawal from alcohol or benzodiazepines [8,9].

The United Kingdom Medicines Control Agency states that bupropion must not be prescribed in patients with other risk factors for seizures, unless there is a compelling clinical justification for which the potential medical benefit of smoking cessation outweighs the potential increased risk of seizure [8]. Predisposing risk factors for seizure include the following: concomitant use of medications known to lower seizure threshold (including antipsychotics, antidepressants, antimalarials, tramadol, theophylline, systemic steroids, quinolones, and sedating antihistamines), history of head trauma, diabetes treated with hypoglycemics or insulin, history of alcohol abuse, and use of stimulants or anorectic products [8].

### The Seizure Risk

Bupropion is associated with a dose-related risk of seizure. The Medicines Control Agency states that the incidence of seizures is one in 1,000 based on doses up to the maximum recommended daily dose of 300 mg per day [8]. The seizure risk may be reduced by taking no more than 150 mg (1 pill) at a time and, if taking two daily doses of 150 mg each (two pills per day), ensuring that doses are taken at least 8 h apart (see www.bnf.org).

Up to 24 July 2002, in the UK there were 184 reports of seizures suspected as being associated with the use of bupropion [8]. In about half of the reports, patients had a past history of seizures and/or risk factors for their occurrence.

### The Presenting Case

In the case we have presented, the patient had no current or previous history of seizure disorders, bulimia, or anorexia nervosa. She was on the sixth day of treatment with bupropion, taking 300 mg per day in two separate doses. She had no other predisposing risk factors for seizure. In particular she was taking no prescription medications, or over-the-counter medications (such as those containing ephedrine products) that are known to lower the seizure threshold. While systemic steroids can lower the seizure threshold, systemic absorption of the nasal steroid drops the patient was taking is an extremely unlikely cause for her seizure (she was not taking a high dose of high-strength drops). Her weight (48 kg) may have been an important factor, since bupropion reaches higher plasma levels in smaller individuals—indeed clinical trials of the drug have generally excluded patients weighing under 100 lb (about 45 kg) [2].

**Learning Points**

- This case report is a useful reminder to clinicians that bupropion is associated with a dose-dependent risk of seizures.
- In about half of the reports of seizure associated with bupropion, there was a past history of seizures and/or risk factors for their occurrence.
- It is extremely important to adequately assess seizure risk before prescribing bupropion.
- Patients should be made aware of the possible adverse effects of the drug.
